# How Followers Differing in Career Motivation Gain Career Profits from Transformational Leaders: A Longitudinal Moderated Mediation Model

**DOI:** 10.3389/fpsyg.2017.01527

**Published:** 2017-09-06

**Authors:** Anja Baethge, Thomas Rigotti, Sylvie Vincent-Hoeper

**Affiliations:** ^1^Work, Organizational, and Business Psychology, Department of Psychology, Johannes Gutenberg-University Mainz Mainz, Germany; ^2^Work and Organizational Psychology, Department of Psychology, University of Hamburg Hamburg, Germany

**Keywords:** transformational leadership, development opportunities, objective career success, subjective career success, career motivation

## Abstract

Although, transformational leadership is among the most thoroughly examined leadership theories, knowledge regarding its association with followers' career outcomes is still limited. Furthermore, the underlying mechanisms explaining how transformational leaders affect their employees' career success are yet not well-understood. Based on theoretical assumptions about the processes involved in setting the goal of “making a career,” we propose an indirect effect of transformational leadership on subjective and objective career success via development opportunities that depends on the level of career motivation of employees. We conducted a longitudinal study with two measurement occasions separated by 13 months with 320 employees of a large IT company. Respondents provided ratings online on their direct supervisor's transformational leadership, their own development opportunities, and career motivation at T1; subjective career success was rated at both time points, whereas objective indicators of career transitions were rated at T2 retrospectively. Using structural equation modeling, we tested the proposed moderated mediation model. The results indicated that transformational leadership increased subordinates' subjective career success via development opportunities. In addition, and contrary to theoretical reasoning, the indirect effect was not significant for employees with high career motivation. Thus, employees high in career motivation appeared not to benefit from the development opportunities offered by transformational leaders. The results are discussed in light of tailored leadership that takes the aspirations, and needs of followers into account.

## Introduction

Transformational leadership is one of the most extensively investigated leadership concepts regarding behavioral and attitudinal outcomes in the work context (Hoch et al., 2017). Transformational leaders inspire and intellectually stimulate their followers and are individually considerate of their followers' needs; these behaviors result in a number of positive effects (Bass, 1999). Recent meta-analyses provide sound evidence for positive relationships between transformational leadership and a wide variety of job outcomes, such as job performance, job satisfaction, commitment, and organizational citizenship behavior (Wang et al., 2011; Hoch et al., 2017). According to Bass (1999), transformational leaders pay attention to the developmental needs of their followers and delegate assignments as opportunities for growth. Thus, a transformational leader is likely to enhance subordinates' skills by offering them development opportunities, which can have a positive effect on their careers.

Although, theoretical considerations suggest a positive relationship between transformational leadership and career success, only a small number of primary studies link transformational leadership with followers' positive career outcomes (cf. Vincent-Höper et al., 2012). There is also a paucity of studies regarding the mechanism linking transformational leadership and employees' career success (e.g., Joo and Lim, 2013). It is established in the literature that followers' resources are built and supported through transformational leadership behavior (Judge and Piccolo, 2004; Lyons and Schneider, 2009). Several studies have provided evidence that the effect of transformational leadership on employees' well-being is indirect, mediated by job resources, such as opportunities for development, meaningfulness, autonomy (e.g., Arnold et al., 2007; Nielsen et al., 2008; Breevaart et al., 2014; Perko et al., 2014), as well as personal resources (e.g., Nielsen and Munir, 2009; Holstad et al., 2014). In addition, it has been shown that the effects of transformational leadership are contingent upon followers' characteristics. Holstad et al. (2014), for instance, demonstrated that the indirect negative relationship between transformational leadership and emotional strain, via social support, is evident only for followers who report high occupational ambition.

To address this lack of research on the question of how transformational leaders affect their followers' career success, this study aims to investigate the underlying mechanisms of the relationship between transformational leadership and subjective as well as objective career success in a longitudinal design. Bringing together prior theorizing and empirical evidence on transformational leadership as well as career research, we propose a moderated mediation model. In line with previous research on the link between transformational leadership and development opportunities (Nielsen et al., 2008), we argue for an indirect effect of transformational leadership on subjective career success via development possibilities. When studying the effects of leadership, the aspirations and needs of the follower cannot be disregarded. Several factors may influence a follower's motivation to climb the career ladder. Based on goal setting theory (Locke and Latham, 1990), we examine the effects of an indicator of goal commitment—i.e., career motivation—on the relationship between transformational leadership and career success via development possibilities. We argue that followers who show high career motivation should profit more from opportunities for development provided by transformational leaders.

Our study makes several contributions to the literature. First, we offer an explanation for the link between transformational leadership and the career success of followers by proposing development possibilities as a potential mediator. Second, we test this effect for both *subjective* and *objective* career success. Third, we follow recent calls to consider the motivational aspects of followers (Holstad et al., 2014) by investigating career motivation as a follower characteristic that may moderate the proposed indirect effect of transformational leadership on career success. Fourth, a key advantage of our study is that we test the underlying mechanisms and boundary conditions simultaneously, drawing on an integrative model. Our study thus contributes to a better understanding of differential effects and offers sound implications for future research as well as personnel development and career guidance with regard to leadership development programs. Finally, the strength of our research design is based on a considerably large sample of professionals, who provided information on transformational leadership, career motivation, development possibilities and both subjective and objective indicators of career success at two distinct time points 1 year apart. The data are analyzed by means of path analyses with inclusion of autoregressors.

### Transformational leadership and career success

Career success can be defined as the positive psychological or work-related outcomes that are the result of work experiences (Judge et al., 1995; Seibert et al., 1999). It can be differentiated into objective and subjective career success. Objective career success describes observable career accomplishments such as promotion, salary, and status (Spurk et al., 2016). Subjective career success refers to an individual's self-evaluation of his/her career progress and his/her career satisfaction (Volmer et al., 2016). The two facets are correlated but not the same (Spurk et al., 2016; Volmer et al., 2016) because one can make visible career advancements without necessarily being satisfied with them. Thus, it is recommended to examine both (c.f. Seibert et al., 1999).

We assume that transformational leadership is related to the objective and subjective career success of followers. The concept of transformational leadership was developed by Burns (1978) and Bass (1985). Transformational leaders are described as fostering the intrinsic motivation of their followers by communicating attractive visions, shared values and common goals. Transformational leadership goes beyond exchange relations and comprises five dimensions (Bass, 1998): *idealized influence (attributed, and behavior), inspirational motivation, intellectual stimulation*, and *individualized consideration*. Idealized influence is displayed by leaders who act as role models by setting an example of the requested behavior (cf. Hobman et al., 2011). Inspirational motivation is demonstrated by leaders who communicate appealing and convincing visions to their followers. By means of intellectual stimulation, employees are called upon to question previous practices and are stimulated to think innovatively. Individual consideration involves the recognition of individual needs and the systematic facilitation of employees' development. As a result of transformational leadership behavior, the followers surrender their own interests for the profit and philosophy of the company and show high involvement and engagement with their work (e.g., Bass, 1990; Bass and Avolio, 1994; Bass et al., 2003).

Bass (1985) states that transformational leadership involves career counseling, recording subordinates' progress, encouraging followers to attend training courses and delegating challenging tasks (subsumed under the category of individualized consideration, e.g., Rafferty and Griffin, 2006). Through coaching and mentoring, transformational leaders meet the needs of their employees and create a supportive environment that allows development to thrive (Bass and Riggio, 2006). In other words, leaders provide development opportunities, defined here as opportunities to develop and practice new skills that might facilitate career advancement. Challenging tasks, on-the-job-training, and the latitude to implement material learned in training courses are examples of development possibilities. In addition, the other dimensions of transformational leadership might help increase the knowledge and skills that are relevant to followers' career. By acting as a role model (idealized influence), by encouraging innovative thinking and new approaches (intellectual stimulation), and by motivating followers to meet the organizational aims (inspirational motivation), leaders are assumed to foster the development of their followers, especially through intellectual stimulation. Transformational leaders provide followers with opportunities to gain competencies and personal development (Sashkin and Rosenbach, 1993; Jung and Sosik, 2002). In line with this reasoning, Nielsen et al. (2008) found, in their cross-sectional study of healthcare workers, a positive correlation between transformational leadership and opportunities for development, which in turn was related to the increased well-being of employees. Sosik et al. (2004) found that the transformational behaviors of mentors were positively related to their protégées' learning orientation and thus their career expectations.

If employees work in a challenging environment and have the opportunity to develop new job-relevant skills, they are likely to improve their career opportunities. In their meta-analysis, Ng et al. (2005) report that training and skill development opportunities were positively related to promotion, salary, and career satisfaction. The delegation of challenging tasks was further identified as a relevant indicator of career success (Korek and Rigotti, 2012; Rohde et al., 2012). Accordingly, Rafferty and Griffin (2006) found, in their cross-sectional sample, that developmental leadership was significantly related to career certainty, role breadth self-efficacy and commitment. Vincent-Höper et al. (2012) analyzed a cross-sectional dataset and found that transformational leadership was positively related to career success. Additionally, two meta-analyses showed a positive relationship between transformational leadership and employees' positive job outcomes (Lowe et al., 1996; Judge and Piccolo, 2004). Based on theoretical assumptions and findings on transformational leadership behavior as well as on the association between development opportunities and occupational success, we postulate that transformational leadership affects followers' occupational success indirectly, through the enhancement of opportunities for development.

However, we assume that not only objective and visible career success but also subjective career success will be increased by the development opportunities transformational leaders offer. First, objective career success should be related to subjective career success (Ng et al., 2005). Employees who make career advancements should also experience subjective career success. Moreover, we expect transformational leaders to increase followers' perception of their own career success. Part of inspirational motivation might be to draw attention to prior success to motivate further efforts. In addition, Ng et al. (2005) found in their meta-analysis that organizational sponsorship (e.g., supervisor support or training and skill development possibilities) is more strongly related to subjective than to objective career success. The authors justify this relationship with the assumption that sponsorship is a form of professional appreciation *per-se* and can be perceived as career success itself (Ng et al., 2005). Transformational leadership could be perceived in that way. We therefore predict the following:

Hypothesis 1: There is a positive indirect relationship between transformational leadership and (a) objective and (b) subjective career success via development opportunities.

### The moderating role of career motivation

In the previous section, we argued that transformational leaders have a positive influence on the career success of their employees because they create working conditions that facilitate development. Adding to the growing body of studies that look at boundary conditions for the impact of transformational leadership on diverse outcomes, we now argue for career motivation as a potential moderator.

As a motivation theory, goal setting theory (Locke and Latham, 1990, 2002) helps us understand why career motivation enhances the effect of (developmental) opportunities—offered by the leader—on career success. The theory states that a goal triggers high performance to the extent that the individual is committed to the goal, invests effort and selects quality strategies. Goal commitment is a function of the importance of the goal (i.e., valence) and of self-efficacy (i.e., expectancy; Locke and Latham, 2002). In our study, we introduce career motivation as an indicator of goal commitment that is related to the goal of striving for career enhancement. We assume that career-motivated employees will value the goal of career success (valence); furthermore, there is empirical evidence that career-motivated have a higher career-related self-efficacy (Day and Allen, 2004). Thus, employees with high career motivation should be willing to use the opportunities offered by transformational leaders to develop and advance (c.f. Buse and Bilimoria, 2014). They should profit more from development opportunities than employees with low career motivation because the former are motivated to use these opportunities.

Similarly, Holstad et al. (2014) find that followers' professional ambition moderates the relationship between supervisory social support (offered by transformational leaders) and follower emotional strain. Employees with high career motivation or professional ambition show a high commitment to work-related goals and strive for professional advancement and promotion (Schaarschmidt and Fischer, 2003; Kieschke and Schaarschmidt, 2008). There is a fit between the motivation of ambitious employees and the aims of transformational leadership. Dvir and Shamir (2003) demonstrate differences in followers' susceptibility to transformational leadership. They propose that subordinates' motivation has an impact on their degree of receptiveness to transformational leadership. According to Abele (1994), career motivation is characterized by the wish to yield excellent results and achieve top positions as well as the willingness to invest as much effort as possible in making good career moves. Based on the person-environment-fit approach (French et al., 1982), we expect opportunities for development to have a stronger impact on career success in cases of high career motivation because the high opportunities fit the employees' (high) need for career advancement. Therefore, we assume that follower career motivation predicts the strength of the indirect effect of opportunities for development in the relation between transformational leadership and followers' career success, as opportunities for development may be of higher relevance for highly motivated followers.

We propose a second-stage moderated mediation as we assume that career-motivated employees will be more willing and able to use their opportunities to attain their career goal success. This leads to the following conditional indirect effect hypothesis:

Hypothesis 2: Career motivation will moderate the strength of the indirect relationship between transformational leadership and (a) objective and (b) subjective career success via development possibilities such that the indirect effect will be higher when career motivation is high (second-stage moderated mediation).

## Methods

### Procedure and sample

In cooperation with a large IT company, a random sample of 3,000 employees was generated and contacted via email. Respondents were asked to take part in an online survey regarding career-related attitudes. The first questionnaire was answered by 1,109 employees, followed by a gap of 13 months before the second one. The final matched sample consisted of 320 employees (56.9% males). We compared the values of all assessed variables at T1 (transformational leadership, development opportunities, career motivation, and career success) and the reported demographic characteristics (gender, age, organizational tenure, full-time employment, length of collaboration with the actual leader, leadership position) between the T2 responders and non-responders via *t*-tests or χ^2^-tests (gender, full-time employment, leadership position). The two groups differed only in organizational tenure, which was higher at T1 among the T2 responders (*M* = 11.3, *SD* = 4.9) than among the non-responders (*M* = 10.5, *SD* = 5.2, *t* = −2.11, *p* = 0.035).

The IT sector is characterized by rapid change, which necessitates continuous education to remain current. Furthermore, large companies offer manifold opportunities for employees to develop or make a career; thus, we considered this sample suitable to address our question. The age of participants ranged from 23 to 56 years, with an average age of 40.9 years (*SD* = 6.8). The organizational tenure ranged from 0 to 27 years, and the average tenure was 11.3 years (*SD* = 4.9). Most participants (78.8%) were employed full-time. The length of collaboration with their current leader ranged from 0 to 27 years, and the average was 2.9 years (*SD* = 2.9). Most participants (69.1%) held no leadership position themselves.

### Measures

#### Transformational leadership

Transformational leadership was assessed with the German translation (Felfe and Goihl, 2006) of the Multifactor Leadership Questionnaire of Bass and Avolio (1995) at T1. We used five subscales (with four items each): Idealized Influence Attributed (IIA), Idealized Influence Behavior (IIB), Individual Consideration (IC), Inspirational Motivation (IM), and Intellectual Stimulation (IS). Example items include the following: “My supervisor instills pride in me for being associated with him/her” (IIA); “My supervisor acts in a way that builds my respect” (IIB); “My supervisor spends time teaching and coaching” (IC); “My supervisor articulates a compelling vision of the future” (IM); and “My supervisor seeks differing perspectives when solving problems.” (IS). Answers were given on a five-point Likert scale ranging from (1) “never” to (5) “regularly/almost always.” The α reliability for the overall scale was 0.96.

Development opportunities. Development possibilities were assessed with four items at T1 (based on items from Martin et al., 1980; Rimann and Udris, 1997; Nübling et al., 2005). An example item is “Does your work allow for development of new skills?” Answers were given on a five-point Likert scale ranging from (1) “strongly disagree” to (5) “strongly agree.” The α reliability was 0.77.

#### Career motivation

Career motivation was assessed at T1 with six items (Abele, 1994). An example item is “I enjoy becoming consistently acquainted with new work tasks.” Participants responded using a seven-point Likert scale ranging from (1) “strongly disagree” to (7) “strongly agree.” The α reliability was 0.90.

#### Subjective career success

Subjective career success was assessed through four items at T1 and T2 (Grebner et al., 2010). An example item is “I made some good career moves.” The answers fell on a seven-point Likert scale ranging from (1) “does not apply at all” to (7) “does apply completely.” The α reliability was 0.78 (T1) and 0.73 (T2).

#### Objective career success

Objective career success was assessed through three items at T2 (cf., Rigotti et al., 2014). We asked participants whether they had experienced a career advancement since T1 (within the last year). In the next step, they were asked which forms of change(s) they went through (multiple answers were possible): “more leadership responsibility,” “more professional responsibilities” and “salary growth.” Answers were (1) yes and (0) no. As it was an index scale, the α reliability was low (0.46).

### Factorial structure of transformational leadership

We ran a confirmatory factor analysis to test the factorial structure of transformational leadership. Table 1 presents the results. The higher-order model (the five sub-dimensions loading on one factor) as well as the five-factor model (five correlated sub-dimensions) showed good fit and had a significantly better fit than the one-factor model (one factors loading on all items). However, the five-factor model also showed a significantly better fit than the higher-order model. As we have no theoretical justification for assuming differential effects of the sub-dimension of transformational leadership, we ran the analyses using the mean of all items. Furthermore, it is suggested to use transformational leadership as an unidimensional concept, as the items are usually highly correlated (Felfe, 2006). In our case, the correlation ranges from 0.62 to 0.84. Additionally, the existence of non-shared variance does not need to mean that there are differential effects of the sub-dimensions. Nevertheless, we ran explorative *post-hoc* analyses of the proposed model for each sub-dimension to test for any possible differential effects.

**Table 1:** Fit Indices of the specified structural models.

**Model**	**χ^2^**	**df**	***SRMR***	***RMSEA***	***90% CI***	***CFI***	***TLI***
A) 5 factors, 1 higher order factor	513.84^***^	165	0.046	0.081	0.073–0.089	0.936	0.927
B) 5 single factors	462.89^***^	160	0.039	0.077	0.069–0.085	0.945	0.934
Difference of A and B	50.95^***^	5					
C) 1 factor	1149.74^***^	170	0.063	0.134	0.127–0.142	0.821	0.800
Difference of B and C	686.85^***^	10					
Difference of A and C	635.91^***^	5					

*SRMR, standardized root mean square residual; RMSEA, root mean square error of approximation; 90% CI, confidence interval for RMSEA; TLI, Tucker–Lewis index; CFI, comparative fit index*.

*** *p < 0.001*.

### Common method variance

As we measured the independent variable (transformational leadership), the mediator (development possibilities), and the moderator (career motivation) at the same time point, common method variance may have biased our results. We, therefore, ran a confirmatory factor analyses, comparing a three-factor model for these constructs with a model where we included an uncorrelated common method factor (see Podsakoff et al., 2012). As could be expected, the model fit improved with the inclusion of a common method factor (Δχ^2^ = 488.91, Δ*df* = 4, *p* < 0.001), but when we compared the variances of the single items of both models (to detect the variance explained by the common method bias), we found that only the items of transformational leadership behavior (range = 0.50–1.16) were above the suggested value of 25% common method variance (Williams et al., 1989). Therefore, we conclude that common method variance is not a substantial bias for our results, as it is not problematic for the distinction of our three focal factors.

### Analyses

We conducted a path model using the R software (version 3.1.2; R Core Team, 2014). We first modeled the main effects that contained the autoregressor of subjective career success (model 1) and then added the mediator variable (model 2) before finally including the moderator and the interaction (model 3). The analyses were based on procedures recommended by Preacher et al. (2007). The model equations were as follows, with *X* = transformational leadership, *M* = development possibilities, *W* = career motivation, *Y1* = subjective career success and *Y2* = objective career success:

Model 1

$$\begin{array}{l} Y1_{T2}=b01+c1{\;}^{*}\;X+d^{*}Y1_{T1}+r\\ Y2_{T2}=b02+c2{\;}^{*}\;X+r \end{array}$$

Model 2

$$\begin{array}{l} M=a0+a1{\;}^{*}\;X+r\\ Y1_{T2}=b01+c'1{\;}^{*}\;X+b1{\;}^{*}\;M+d{\;}^{*}\;Y1_{T1}+r\\ Y2_{T2}=b02+c'2{\;}^{*}\;X+b4{\;}^{*}\;M+r \end{array}$$

Model 3

$$\begin{array}{l} M=a0+a1{\;}^{*}\;X+r\\ Y1_{T2}=b01+c'1{\;}^{*}\;X+(b1+b3{\;}^{*}\;W){\;}^{*}\;M+b2{\;}^{*}\;W\\ \;+\;d{\;}^{*}\;Y1_{T1}+r\\ Y2_{T2}=b02+c\text{'}2{\;}^{*}\;X+(b4+b6{\;}^{*}\;W){\;}^{*}\;M+b5{\;}^{*}\;W+r. \end{array}$$

The intercepts, as well as the covariances between all T1-variables, were freely estimated, with an exception being the path between *X* and *M*. We also allowed a covariance between the dependent variables *Y1* and *Y2*. The independent variables were standardized. The models were bootstrapped with 10,000 draws. The indirect effect of *X* on *Y1*(*Y2*) was defined as â*1* * $\hat{b}$*1(â1* * $\hat{b}$*4)* (model 2). The conditional indirect effect of *X* on *Y* was â*1* * ($\hat{b}$*1* + $\hat{b}$*3* * *W) (â1* * ($\hat{b}$*4* + $\hat{b}$*6* * *W)* (model 3). We calculated the conditional indirect effect at specified levels of *W* (0, +1 *SD* and −1 *SD*). Furthermore, we calculated the index of the conditional indirect effect (â*1* ^*^ *b3* for subjective career success and â*1* ^*^ *b6* for objective career success).

## Results

The means, standard deviations, and intercorrelations of all variables are displayed in Table 2.

**Table 2:** Means, standard deviations, and correlations among study variables (*N* = 320 employees).

		***M***	***SD***	**1**	**2**	**3**	**4**	**5**
1	Transformational leadership	3.28	0.97					
2	Development possibilities	3.27	0.80	0.29^***^				
3	Career motivation	5.89	0.93	0.27^***^	0.08			
4	Subjective career success (t1)	2.85	1.39	0.22^***^	0.23^***^	0.39^***^		
5	Subjective career success (t2)	2.77	1.29	0.14^*^	0.29^***^	0.18^**^	0.48^***^	
6	Objective career success (t2)	0.67	0.17	0.03	0.07	−0.05	−0.02	0.20^***^

t1, time point 1; t2, time point 2;

* *p < 0.05*.

** *p < 0.01*.

*** *p < 0.001*.

Table 3 gives an overview of the results of the path models. Subjective and objective career success were not directly predicted by transformational leadership (subjective career success: β = 0.05, *p* = 0.554; objective career success: β = 0.01, *p* = 0.718). Thus, Hypothesis 1 proposed an indirect effect of transformational leadership on (a) subjective and (b) objective career success via developmental possibilities. The indirect effect was significant for subjective career success (*c* = 0.08, *p* = 0.001) and not significant for objective career success (*c* = 0.00, *p* = 0.368), indicating that Hypothesis 1a was supported, whereas Hypothesis 1b must be rejected.

**Table 3:** Results of the path models of career success (*N* = 320).

	**Model 1**		**Model 2**		**Model 3**	
	**β**	***SE***	**β**	***SE***	**β**	***SE***
**DEPENDENT VARIABLE: SUBJECTIVE CAREER SUCCESS (t2)**						
Intercept	2.77^***^	0.06	2.77^***^	0.10	2.79^***^	0.06
Career success (t1)	0.61^***^	0.07	0.57^***^	0.07	0.58^***^	0.07
Transformational leadership	0.05	0.08	−0.02	0.07	0.04	0.07
Development opportunities			0.26^***^	0.07	0.26^***^	0.06
Career motivation					0.04	0.08
DO × CM					−0.22^**^	0.07
**DEPENDENT VARIABLE: OBJECTIVE CAREER SUCCESS (t2)**						
Intercept	0.07^***^	0.01	0.07^***^	0.01	0.07^***^	0.01
Transformational leadership	0.01	0.01	0.00	0.02	0.01	0.01
Development opportunities			0.01	0.01	0.01	0.01
Career motivation					0.00	0.01
DO × CM					−0.04^*^	0.02
**DEPENDENT VARIABLE: DEVELOPMENT OPPORTUNITIES**						
Transformational leadership			0.29^***^	0.07	0.29^***^	0.07

*t1, time point 1; t2, time point 2; DO × CM, Interaction of development opportunities and career motivation*.

* p < 0.05;

** p < 0.01;

*** *p < 0.001*.

**Figure 1:**
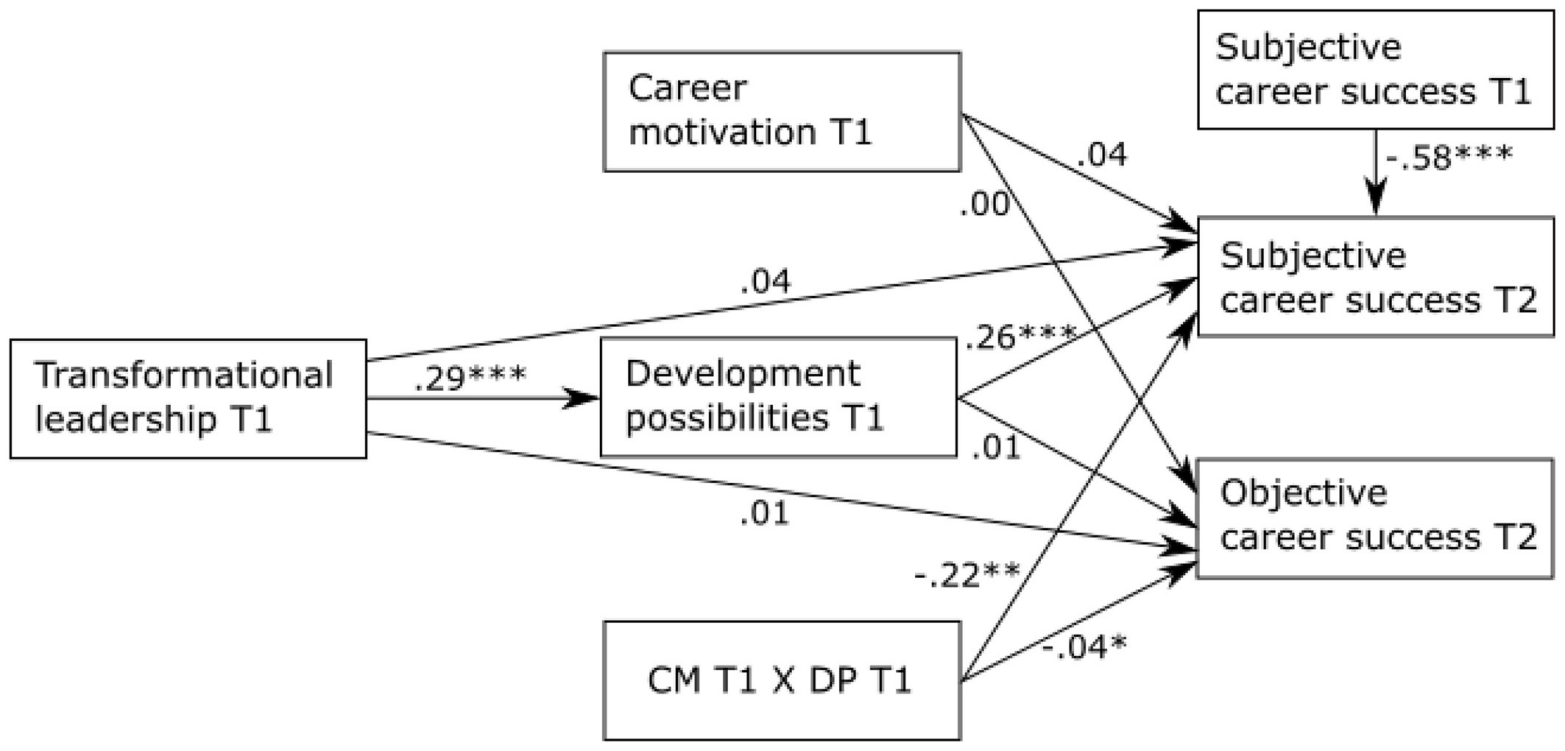
The conditional indirect effect model. CM, career motivation; DP, development possibilities; T1, time point 1; T2, time point 2. The values are standardized regression coefficients. ^*^p < 0.05; ^**^p < 0.01; ^***^p < 0.001.

**Figure 2:**
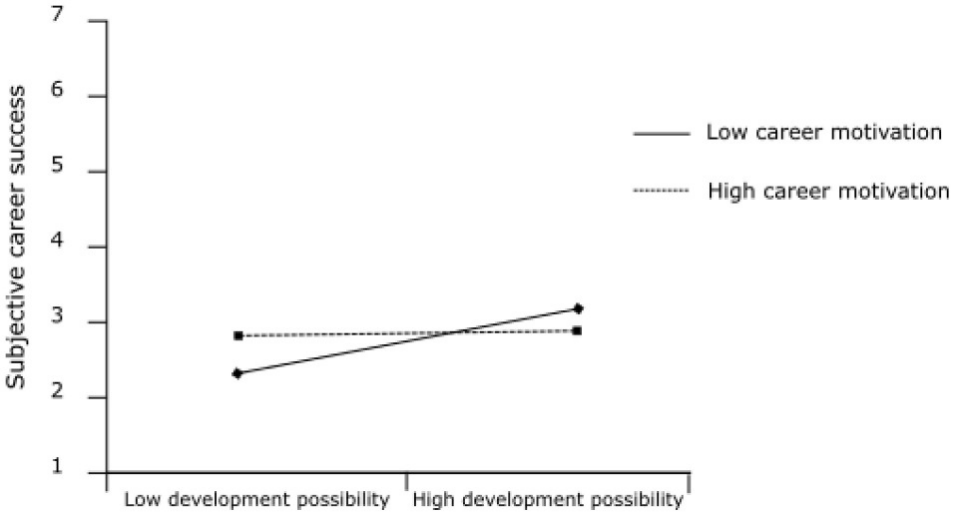
The interaction of career motivation and development possibilities in predicting subjective career success.

**Figure 3:**
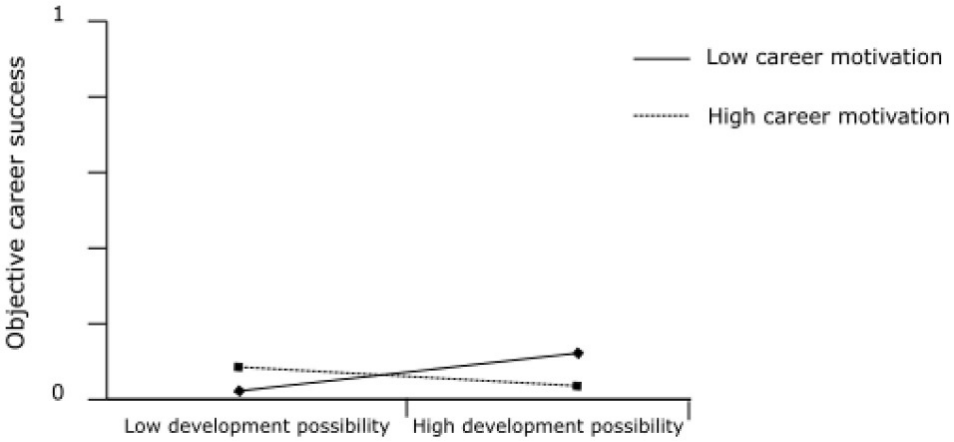
The interaction of career motivation and development possibilities in predicting objective career success.

In Hypothesis 2, we proposed that career motivation moderates the indirect relationship of transformational leadership with (a) subjective career success and (b) objective career success via development possibilities such that the indirect effect is stronger when career motivation is high than when it is low. Figure 1 shows the results of the moderated mediation analysis. The interaction of career motivation and development possibilities and the index of the conditional indirect effect are significant for both outcomes. Figure 2 depicts the interaction effect of career motivation and development possibilities (simple slope tests: +1 *SD*: *t* = 0.34, *p* = 0.734; −1 *SD*: *t* = 3.92, *p* < 0.001) on subjective career success. Figure 3 depicts the interaction effect of career motivation and development possibilities (simple slope tests: +1 *SD*: *t* = −0.16, *p* = 0.876; −1 *SD*: *t* = 0.33, *p* = 0.741) on objective career success.

Table 4 provides an overview of the results of the tests of the specified conditional indirect effects (cie). In the case of subjective career success, there are significant conditional indirect effects for low and average values of career motivation but not for high career motivation (−1 *SD*: *cie* = 0.14, *p* < 0.001; 0: *cie* = 0.08, *p* = 0.002; +1 *SD*: *cie* = 0.01, *p* = 0.625). In the case of objective career success, there is a marginal significant conditional indirect effect for low career motivation but not for average or high values of career motivation (−1 *SD*: *cie* = 0.01, *p* = 0.057; 0: *cie* = 0.00, *p* = 0.295; +1 *SD*: *cie* = −0.01, *p* = 0.151). This conditional indirect effect is in contrast to the anticipated effects proposed in H3.

**Table 4:** Conditional indirect effects of transformational leadership on career success (*N* = 320).

**Defined values of the moderator**	**Conditional indirect effect**
**SUBJECTIVE CAREER SUCCESS**	
Career motivation = −1 *SD*	0.139^***^
Career motivation = 0	0.076^*^
Career motivation = +1 *SD*	0.014
Index of the conditional indirect effect	−0.063^**^
**OBJECTIVE CAREER SUCCESS**	
Career motivation = −1 *SD*	0.014^+^
Career motivation = 0	0.004
Career motivation = +1 *SD*	−0.007
Index of the conditional indirect effect	−0.011^*^

+ p < 0.1;

* p < 0.05;

** p < 0.01;

*** *p < 0.001*.

### Additional analyses of the three indicators of objective career success

To test which of the indicators of objective career success (leadership advancement, salary increase, increase in professional responsibilities) contributed to the effect, we conducted (single) *post-hoc* analyses for each facet. Because they were binomial variables, we had to use the DWLS estimator and define the outcomes as ordered (cf. Lavaan Tutorial, 2016). Table 5 gives an overview of the results of the path models and the tests of the specified conditional indirect effects. Leadership advancement was shown to have the strongest effects. There was a significant interaction effect (*c* = −0.22, *p* = 0.011), and the conditional indirect effect was significant in the case of low motivation but not in the cases of high and medium motivation (−1 *SD*: *cie* = 0.14, *p* = 0.023; 0: *cie* = 0.07, *p* = 0.147; +1 *SD*: *cie* = 0.01, *p* = 0.890). The same pattern was found for salary increase, but the conditional indirect effect was only marginally significant (interaction: *c* = −0.22, *p* = 0.028; −1 *SD*: *cie* = 0.11, *p* = 0.093; 0: *cie* = 0.04, *p* = 0.401; +1 *SD*: *cie* = −0.02, *p* = 0.679). There were no significant effects for the increase in professional responsibilities (interaction: *c* = −0.13, *p* = 0.173 −1 *SD*: *cie* = 0.04, *p* = 0.345; 0: *cie* = 0.00, *p* = 0.903; +1 *SD*: *cie* = −0.03, *p* = 0.394).

**Table 5:** Results of the *post-hoc* tests (***N*** = 320).

	**Leadership advancement**		**Professional responsibility**		**Salary increase**	
	**β**	***SE***	**β**	***SE***	**β**	***SE***
**DEPENDENT VARIABLE: OBJECTIVE CAREER SUCCESS INDICATOR (t2)**						
Intercept	0.00	0.03	0.00	0.04	0.01	0.03
Transformational leadership	0.04	0.17	0.06	0.11	0.15	0.16
Development opportunities	0.24	0.16	0.01	0.10	0.14	0.16
Career motivation	−0.03	0.16	0.00	0.10	−0.08	0.14
DO × CM	−0.22^*^	0.09	−0.13	0.10	−0.22^*^	0.10
**DEPENDENT VARIABLE: DEVELOPMENT OPPORTUNITIES**						
Transformational leadership	0.29^***^	0.06	0.29^***^	0.07	0.29^***^	0.07
*Conditional indirect effects*						
Career motivation = −1 *SD*	0.14^*^	0.06	0.04	0.04	0.11^+^	0.06
Career motivation = 0	0.07	0.05	0.00	0.03	0.04	0.05
Career motivation = +1 *SD*	0.01	0.06	−0.03	0.04	−0.02	0.06
Index of the conditional indirect effect	−0.06^*^	0.03	−0.04	0.03	−0.07^+^	0.04

t1, time point 1; t2, time point 2; DO × CM, Interaction of development opportunities and career motivation

+ p < 0.1;

* p < 0.05;

*** *p < 0.001*.

### *Post-hoc* analyses of the five sub-dimensions of transformational leadership

As the CFA showed that transformational leadership consists of five sub-dimensions, we ran additional analyses to test whether these sub-dimensions led to different results. We ran the proposed moderated mediation model for each subscale. Table 6 shows the conditional indirect effects for each sub-facet, and they all led to literally the same effects.

**Table 6:** Conditional indirect effects of the sub-dimensions of transformational leadership on career success (*N* = 320).

	***Iia***	***Iib***	***Ic***	***Im***	***Is***
**DEPENDENT VARIABLE: SUBJECTIVE CAREER SUCCESS (t2)**					
Career success (t1)	0.58^***^	0.58^***^	0.58^***^	0.58^***^	0.57^***^
Transformational leadership	−0.00	0.04	0.04	0.02	0.06
Development opportunities	0.27^***^	0.26^***^	0.26^***^	0.27^***^	0.26^***^
Career motivation	0.05	0.04	0.04	0.04	0.04
DO × CM	−0.21^**^	−0.21^**^	−0.22^**^	−0.21^**^	−0.22^**^
**DEPENDENT VARIABLE: OBJECTIVE CAREER SUCCESS (t2)**					
Transformational leadership	0.01	0.01	0.01	0.01	0.02
Development opportunities	0.02	0.02	0.01	0.01	0.01
Career motivation	0.00	0.00	0.00	0.00	0.00
DO × CM	−0.04^*^	−0.04^*^	−0.04^*^	−0.04^*^	−0.04^*^
**DEPENDENT VARIABLE: DEVELOPMENT OPPORTUNITIES**					
Transformational leadership	0.25^***^	0.25^***^	0.31^***^	0.25^***^	0.24^***^
**CONDITIONAL INDIRECT EFFECTS FOR SUBJECTIVE CAREER SUCCESS**					
Career motivation = −1 *SD*	0.117^**^	0.120^**^	0.148^***^	0.120^**^	0.115^***^
Career motivation = 0	0.066^**^	0.066^**^	0.081^**^	0.067^**^	0.062^**^
Career motivation = +1 *SD*	0.016	0.012	0.014	0.014	0.009
Index of the conditional indirect effect	−0.051^**^	−0.054^**^	−0.067^**^	−0.053^**^	−0.053^**^
**CONDITIONAL INDIRECT EFFECTS FOR OBJECTIVE CAREER SUCCESS**					
Career motivation = −1 *SD*	0.012^+^	0.012^+^	0.015^+^	0.012^+^	0.012^+^
Career motivation = 0	0.004	0.004	0.004	0.003	0.003
Career motivation = +1 *SD*	−0.005	−0.005	−0.007	−0.005	−0.006
Index of the conditional indirect effect	−0.009^*^	−0.009^*^	−0.011^*^	−0.009^*^	−0.009^*^

*Reported are the β-weights and the conditional indirect effects. Iia, Idealized influence attributed; Iib, Idealized influence behavior; Ic, Individual consideration; Im, Inspirational motivation; Is, Intellectual stimulation*.

+ p < 0.1;

* p < 0.05;

** p < 0.01;

*** *p < 0.001*.

## Discussion

The aim of the study was to test a conditional indirect effects model for the relationship between transformational leadership and subjective and objective career success. Developmental opportunities were argued to be an important linking mechanism in this respect, which our results validated for subjective career success. Furthermore, both indirect effects were found to be conditional upon career motivation. Surprisingly, employees with lower career motivation appear to profit more from transformational leaders in terms of their own subjective and objective career success. *Post-hoc* analyses showed that the effect on objective success is mainly based on advancements in the leadership position. Furthermore, the effects of transformational leadership show the same pattern for each sub-dimension.

We showed that transformational leadership promotes the (mainly subjective) career success of subordinates by offering developmental opportunities. Of course, other mediating mechanisms are possible, such as increasing chances of performance accomplishments (Xanthopoulou et al., 2008; cf. Si and Wei, 2012; Lin et al., 2016; Losch et al., 2016). However, whereas attaining goals often means attaining the goals of the leader or the company, developmental opportunities focus directly on the needs of the employee. In our study, we wanted to understand the role of transformational leaders with respect to the individual needs of employees (making a career, developing new skills). We also ran the proposed model controlling for performance, and the results stayed the same (subjective career success: *c* = 0.07, *p* = 0.003; objective career success: *c* = 0.00, *p* = 0.359). Thus, offering developmental opportunities plays an important role in fostering the career of subordinates (beyond the effect of increased performance).

Despite the manifold positive effects of transformational leadership (Judge and Piccolo, 2004), there is growing evidence concerning an ambivalent role of transformational leadership (e.g., Kark et al., 2003; Tourish, 2014). For example, Anderson and Sun (2015) reported that followers of less transformational leaders engage more in networking behaviors, which is considered an important predictor for career success (Seibert et al., 2001; Wolff and Moser, 2009). Concerning the moderating effect of career motivation, it appears that employees with high career motivation do not need to rely on their transformational leaders. Rather, they demonstrate more proactive behavior in reaching their career goals. The positive link between career motivation and subjective career success supports this reasoning.

As transformational leadership was found to have a weak but substantial positive relationship with career motivation, we cannot argue for a general erosion of career motivation under the leadership of transformational supervisors. The opposite seems to be the case, in that transformational leaders seem to enhance career motivation in the first place, and career motivation is related to career success (Table 2). However, does the moderating effect of career motivation contradict propositions put forward by goal setting theory? According to goal setting theory, career motivation should be helpful in reaching the aim of making a career. Hence, career motivation (as proxy of goal commitment in this case) might facilitate career progress but not by every means. It is possible that career-motivated employees choose another way to get ahead. Our results show that career-motivated employees do not profit from development possibilities. One could argue that such (external) assistance could even have an overjustification effect (Deci and Ryan, 1985; Cameron and Pierce, 1994; Tang and Hall, 1995). If developmental opportunities, such as special tasks, given to develop an employee's skills are interpreted as external control (i.e., the leader has assigned me the task because he or she wants me to reach a certain goal), the follower's sense of autonomy and self-determination declines, which in turn decreases (intrinsic) motivation. If this reasoning is true, the leader should take care not to undermine the autonomy and self-determination of intrinsically career-motivated employees through well-intentioned tasks/demands. A similar explanation is offered by the theoretical approach on the visibility of social support by Bolger and Amarel (2007). They state that visible (and unasked) social support might have detrimental effects on self-worth, as it can be interpreted as sign of incompetence of the recipient. Because support from the leader is very visible, and because providing developmental opportunities might be interpreted as indicating a need for development, this kind of support might have also detrimental effects on career-motivated employees, who might already take care of their skill development on their own. Concordant with that, Crockett et al., 2017 also found that visible social support is especially detrimental for people with high self-efficacy. A somewhat related explanation is that career-motivated employees possibly do not get ahead by developing even more new skills but rather by networking, organizational politics (Seibert et al., 2001; Wolff and Moser, 2009) or other strategies. Thus, our results do not necessarily contradict goal setting theory; rather, they show that it is necessary to ask which means help which employees reach their aims. Career-motivated employees may differ not only in their motivation but also in their preconditions in skills, self-efficacy and thus career-related needs. There is clearly a need for further research to disentangle these different *post-hoc* explanations. Nevertheless, our study contributes to existing research by showing that transformational leadership can help employees achieve their individual career goal but characteristics of followers need to be taken into account. Career-motivated employees seem to be a special case, as they do not profit from developmental opportunities. Further research is needed to disentangle whether there are more means (beyond providing developmental opportunities) used by transformational leaders that are not beneficial for career-motivated employees. Additional analyses of our data showed that developmental opportunities are the crucial point. Transformational leadership increases both objective and subjective career success the more career-motivated an employee is (interaction effect of transformational leadership and career motivation: Subjective career success: β = 0.14, *p* < 0.001; objective career success: β = 0.02, *p* < 0.001).

Another interesting result is the different effects found for subjective and objective career success. Consistent with previous research, they are just moderately correlated (c.f. Spurk et al., 2016; Volmer et al., 2016). Therefore, objective and subjective career success obviously do not measure the same constructs, as people who are objectively successful do not necessarily evaluate this success as subjective career success, and vice versa (Seibert et al., 1999). Furthermore, career advancements might exist that are independent of attaining a higher leadership position, more money or more professional responsibilities, e.g., receiving tasks that are more visible within the company or collaborating with important people. We want to note that the pure mediation effect was found only for subjective career success and not for objective career success. This is in line with the ideas of Ng et al. (2005), who stated that subjective career success can also be experienced because of sponsorship, which is perceived as professional appreciation. When transformational leadership leads to more development possibilities, this could be perceived as professional appreciation and thus lead to the experience of subjective career success. The mediation effect was significant only for employees with low career motivation in the case of objective career success. Thus, employees with low career motivation, who possibly do need a lot of assistance, can objectively profit from the efforts of the transformational leader to get ahead (attain a leadership position). Others might perceive it as appreciation (good intent) but as having no effect on their objective career outcomes. Perhaps too much help can undermine the autonomy of highly career-motivated followers.

The results of the confirmatory factor analysis suggested that the sub-dimensions of transformational leadership can meaningfully be separated, with high correlations between the five facets. Therefore, we also ran a set of analyses testing the single sub-dimensions of transformational leadership in our research model. The results indicated literally no differences in the effects between the sub-dimensions. Hence, in our case, treating transformational leadership as a unidimensional concept seems to be justified. None of the sub-facets seems to play a special role in relation to offering developmental opportunities and its consequences.

## Limitations and strengths

This study has several strengths. First, the longitudinal design enabled us to assess predictors and criteria at different points in time. Furthermore, we controlled for the autoregressor at T1 of the criteria of subjective career success, and objective career success was measured with a direct question of change within the last year. Therefore, we actually measured career *advancement*, a change over time in subjective and objective indicators of career success. Moreover, we included both outcomes in one model to reduce Type I errors.

Nevertheless, several methodological issues need to be accounted for when interpreting our results. First, the indicators were assessed by the same source: followers. Objective career success was at least examined with unambiguous questions, which hopefully measured an objective state, but other sources would possibly be more objective. Career motivation and subjective career success need to be assessed by the individual, but developmental opportunities could be examined using objective parameters. Transformational leadership could be assessed by the leader or by the whole team, which would provide interesting angles for multilevel models.

Moreover, we examined the mediation model with two time points. Thus, we were only able to separate the *outcome* from the independent variable and the mediator in time. Three time points would be methodologically better. We cannot test whether transformational leadership really precedes developmental opportunities. However, when we reflect on the process of transformational leaders promoting their subordinates, there is no reason to assume that leaders are first experienced as transformational and then give developmental opportunities. Both experiences/actions presumably occur simultaneously. (A transformational leader is experienced as transformational because of the behavior the leader shows, e.g., giving developmental opportunities.) However, the step from supervisor support to career success takes some time. Thus, with regard to content, it makes sense to ask this question just at two time points.

Furthermore, we had a highly homogenous sample of employees of one international IT company. The IT sector is characterized by rapid changes. Thus, the development of new skills might be of special importance not only for employees to advance but also to continue to work in their current position. Whether our mediation effect of development possibilities can be replicated in less dynamic fields needs to be examined. As of now, our results may only be generalizable to knowledge workers.

### Implications for practice

Despite its limitations, our study offers a set of practical implications. It is evident that providing developmental opportunities is a good way to enhance the career success of followers. This is the case especially for employees with low or medium career motivation. Employees with high career motivation seem to need other forms of support. They do not profit from developmental opportunities. Based on the research on detrimental effects of visible social support (Bolger and Amarel, 2007) or overjustification effects (Deci and Ryan, 1985; Cameron and Pierce, 1994; Tang and Hall, 1995), it is recommended to be careful with providing assistance for that special group, as it may compromise their feeling of self-determination. Increasing the visibility or autonomy of highly career-motivated employees could be a good alternative to support their career advancement.

## Conclusions

Our study contributes to a more differentiated perspective on the positive effects of transformational leadership. There are risks involved in transformational leadership when followers' motivation is neglected. Especially for followers with a high intrinsic career motivation, transformational leadership may undermine the internal locus of control and needs for autonomy. Taking team members by the hand at every step may hinder their self-development and their feeling of personal accomplishment. Development opportunities should thus be negotiated and offered in a way that ensures that followers can attribute success to their own proactive behavior.

## Ethics statement

We followed research procedures in accordance with the Helsinki Declaration, as revised in 2013. An ethics approval was not required as per institutional guidelines and national laws. Our research was promoted by the Federal Ministry of Education and Research in Germany. Contact with study participants was established via their employers. Consent for the study was given by local work councils. All participants in our study were provided sufficient information in order to be able to give informed consent (by following the invitation to fill in the questionnaires) to take part in this study. Research respondents were ensured confidentiality and anonymity. All participation was voluntary, and we informed participants that they could withdraw from the study at any time without explanation. After receiving the information on the voluntariness and procedures of the study, participants gave their consent to participate by completing the survey. Reports on the general results of the study were made available to all participants. We confirm that this research is independent and impartial.

## Author contributions

AB, TR, and SV substantially contributed to the development of the present manuscript. TR and SV were involved in the planning and execution of the data collection. TR, AB, and SV developed the theoretical background. AB and TR primarily derived the hypotheses. AB analyzed the data. AB and TR were involved in the interpretation of the results. AB, TR, and SV were concerned with drafting the work and revising it critically.

### Conflict of interest statement

The authors declare that the research was conducted in the absence of any commercial or financial relationships that could be construed as a potential conflict of interest.
